# Preclinical Investigation of Trifluoperazine as a Novel Therapeutic Agent for the Treatment of Pulmonary Arterial Hypertension

**DOI:** 10.3390/ijms22062919

**Published:** 2021-03-13

**Authors:** Yann Grobs, Charifa Awada, Sarah-Eve Lemay, Charlotte Romanet, Alice Bourgeois, Victoria Toro, Valérie Nadeau, Kana Shimauchi, Mark Orcholski, Sandra Breuils-Bonnet, Eve Tremblay, Steeve Provencher, Roxane Paulin, Olivier Boucherat, Sébastien Bonnet

**Affiliations:** 1 Pulmonary Hypertension Research Group, Québec Heart and Lung Institute Research Centre, Québec City, QC G1V 4G5, Canada; yann.grobs.1@ulaval.ca (Y.G.); charifa.awada.1@ulaval.ca (C.A.); sarah-eve.lemay@criucpq.ulaval.ca (S.-E.L.); charlotte.romanet.1@ulaval.ca (C.R.); alice.bourgeois.2@ulaval.ca (A.B.); victoria.toro@criucpq.ulaval.ca (V.T.); valerie.nadeau@criucpq.ulaval.ca (V.N.); kana.shimauchi@criucpq.ulaval.ca (K.S.); mark.orcholski@criucpq.ulaval.ca (M.O.); sandra.breuils-bonnet@criucpq.ulaval.ca (S.B.-B.); eve.tremblay@criucpq.ulaval.ca (E.T.); Steeve.Provencher@criucpq.ulaval.ca (S.P.); roxane.paulin@criucpq.ulaval.ca (R.P.); olivier.boucherat@criucpq.ulaval.ca (O.B.); 2 Department of Medicine, Laval University, Québec City, QC G1V 4G5, Canada

**Keywords:** vascular remodeling, FOXO3, autophagy, smooth muscle cells, drug repositioning

## Abstract

Trifluoperazine (TFP), an antipsychotic drug approved by the Food and Drug Administration, has been show to exhibit anti-cancer effects. Pulmonary arterial hypertension (PAH) is a devastating disease characterized by a progressive obliteration of small pulmonary arteries (PAs) due to exaggerated proliferation and resistance to apoptosis of PA smooth muscle cells (PASMCs). However, the therapeutic potential of TFP for correcting the cancer-like phenotype of PAH-PASMCs and improving PAH in animal models remains unknown. PASMCs isolated from PAH patients were exposed to different concentrations of TFP before assessments of cell proliferation and apoptosis. The in vivo therapeutic potential of TFP was tested in two preclinical models with established PAH, namely the monocrotaline and sugen/hypoxia-induced rat models. Assessments of hemodynamics by right heart catheterization and histopathology were conducted. TFP showed strong anti-survival and anti-proliferative effects on cultured PAH-PASMCs. Exposure to TFP was associated with downregulation of AKT activity and nuclear translocation of forkhead box protein O3 (FOXO3). In both preclinical models, TFP significantly lowered the right ventricular systolic pressure and total pulmonary resistance and improved cardiac function. Consistently, TFP reduced the medial wall thickness of distal PAs. Overall, our data indicate that TFP could have beneficial effects in PAH and support the view that seeking new uses for old drugs may represent a fruitful approach.

## 1. Introduction

Pulmonary arterial hypertension (PAH) is a complex and life-threatening condition clinically defined as a mean pulmonary artery (PA) pressure greater than 20 mmHg at rest [1]. At the physiological level, PAH is characterized by chronic vasoconstriction and progressive PA wall thickening, mainly due to unbridled proliferation and apoptosis evasion of PA smooth muscle cells (PASMCs) [2,3]. As the obliterative vascular remodeling progresses, increasing pulmonary vascular resistance occurs, leading to right ventricular dysfunction and premature death [4]. Although significant strides have been made in the treatment of PAH, approved drugs that primarily address the vasoconstrictive phenotype of the disease only offer a limited benefit in terms of morbidity and mortality [5], warranting the need to develop new therapeutic strategies mainly devoted to combat the pulmonary vascular remodeling process.

Over the past ten years, significant efforts have been made to decipher the molecular mechanisms governing the cancer-like behavior of PASMCs. Indeed, it is now well accepted that in response to pro-inflammatory cytokines and growth factors such as interleukin 6 (IL-6), endothelin 1 (ET1) and platelet growth factor BB (PDGF-BB) released by stressed PA resident cells, PASMCs progressively develop a hyperproliferative and apoptosis-resistant phenotype. Thanks to the advances in cancer research, a broad array of intracellular factors have now been identified to be dysregulated in disease cells. For instance, constitutive activation of interconnected signaling pathways including mitogen-activated protein kinase (MAPK)/extracellular signal-regulated kinase (ERK) and phosphatidylinositol 3-kinase (PI3K)/AKT signaling along with increased expression and/or activity of multiple transcription factors have been documented impacting different biological pathways such as DNA damage response, cell metabolism and autophagy, which, in turn, act synergistically to enhance PASMC contractility, proliferation and resistance to apoptosis [2,6,7,8]. In this regard, simultaneous targeting of distinct mechanisms might be of particular interest.

Besides the development of new compounds, drug repurposing/repositioning has become an attractive strategy to treat cancer and PAH [9,10], taking advantage of the well-known safety and pharmacokinetics profiles of drugs already in use for a disease to treat another illness and, thus, expedite therapies to the clinic. The antipsychotic drug trifluoperazine (TFP) was recently repurposed for cancer treatment due to its anti-tumor activity in various preclinical models [11,12,13,14,15]. Although the exact mechanism of action of TFP remains unclear, TFP was initially shown to inhibit dopamine receptors and functions as an antagonist of calmodulin, a key regulator of calcium-dependent signal transduction critically implicated in vascular smooth muscle cell contraction and proliferation [16,17]. Further studies revealed that TFP reduces tumor cell proliferation and induces tumor cell apoptosis by targeting various signaling pathways. Notably, TFP was reported to inhibit the AKT/forkhead box protein O3 (FOXO3) axis and to interfere with DNA repair mechanisms and autophagy [13,15,18,19], all implicated in the abnormal behavior of PAH-PASMCs. However, whether TFP has a therapeutic effect on PAH remains unknown. To address this question, we investigated the in vitro effects of TFP on PAH-PASMCs and tested its therapeutic potential in two complementary and well-established animal models mimicking the disease.

## 2. Results

### 2.1. TFP Significantly Reduced PAH-PASMC Proliferation and Survival

**Figure 1:**
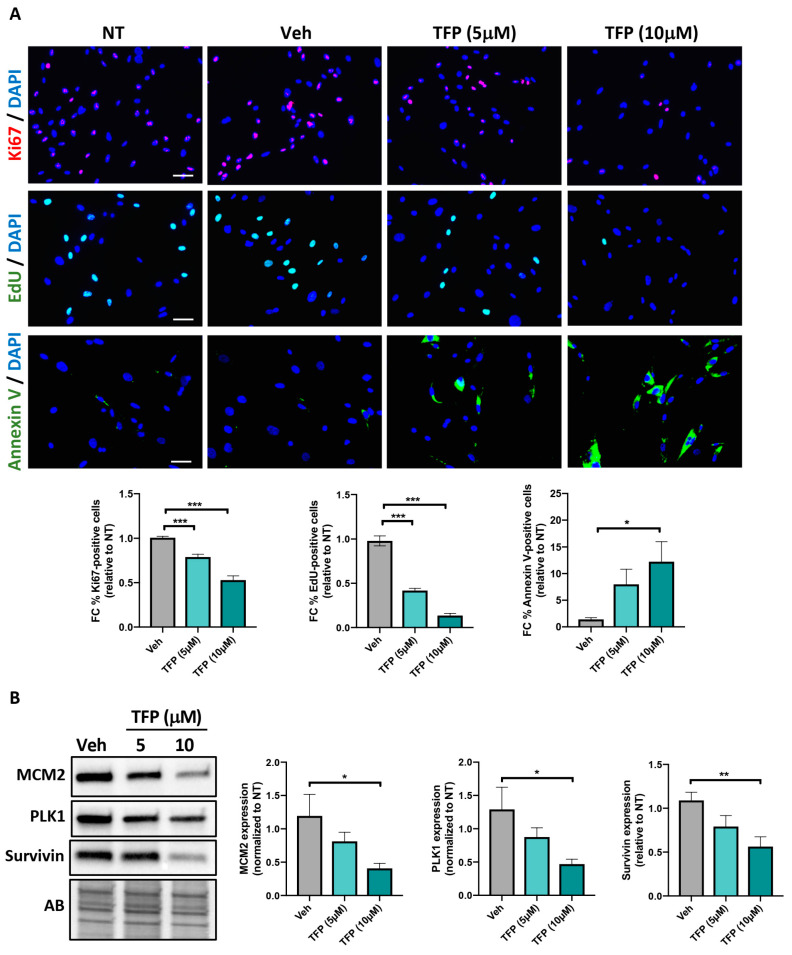
Trifluoperazine (TFP) elicits anti-proliferative and anti-pro-survival effects on pulmonary arterial hypertension pulmonary artery smooth muscle cells (PAH-PASMCs). (**A**) Proliferation (Ki67 and 5-Ethynyl-2′-deoxyuridine (EdU)) and apoptosis (Annexin V) were measured in PAH-PASMCs after treatment or not with TFP or its vehicle (Veh, DMSO) for 24 h. Representative immunofluorescence images of Ki67-, EdU- and Annexin V-positive cells as well as corresponding quantifications are shown. (**B**) Representative Western blots and corresponding densitometric analyses of mini chromosome maintenance protein 2 (MCM2), polo-like kinase 1 (PLK1) and Survivin in PAH-PASMCs exposed or not to the indicated concentration of TFP or its vehicle for 24 h. Data are expressed as fold change (FC) relative to untreated cells and presented as mean ± SEM. Protein expression was normalized to Amido black (AB). Experiments were performed in triplicate in at least four different PAH-PASMC cell lines. * *p* < 0.05; ** *p* < 0.01 and *** *p* < 0.001. Scale bars: 50 μm.

To determine whether TFP elicits therapeutic effects in vitro, PAH-PASMCs were exposed to two different concentrations (5 and 10 μM) of TFP for 24 h. We first analyzed its impact on cell proliferation by measuring the percentage of PAH-PASMCs exhibiting nuclear expression of Ki67. As shown in Figure 1A, a significant dose-dependent decrease in the percentage of Ki-67-labeled PAH-PASMCs was observed upon TFP exposure. PAH-PASMC proliferative capacity was then assessed by 5-Ethynyl-2′-deoxyuridine (EdU) incorporation assay. Consistently, treatment with TFP resulted in a dose-dependent slower EdU incorporation rate compared to vehicle-treated cells (Figure 1A). Having demonstrated that TFP decreases PAH-PASMC proliferation, we sought to determine whether the compound has anti-survival effects. We found that TFP significantly increases cell death, as revealed by Annexin V staining. In agreement with these data, expression levels of mini chromosome maintenance protein 2 (MCM2) and polo-like kinase 1 (PLK1) (two cell proliferative markers) and Survivin (an anti-apoptosis factor) were dose-dependently diminished in TFP-treated PAH-PASMCs (Figure 1B). Our results indicate that TFP exerts anti-proliferative and anti-survival effects on PAH-PASMCs.

### 2.2. Effects of TFP on AKT/FOXO3 Signaling in PAH-PASMCs

**Figure 2:**
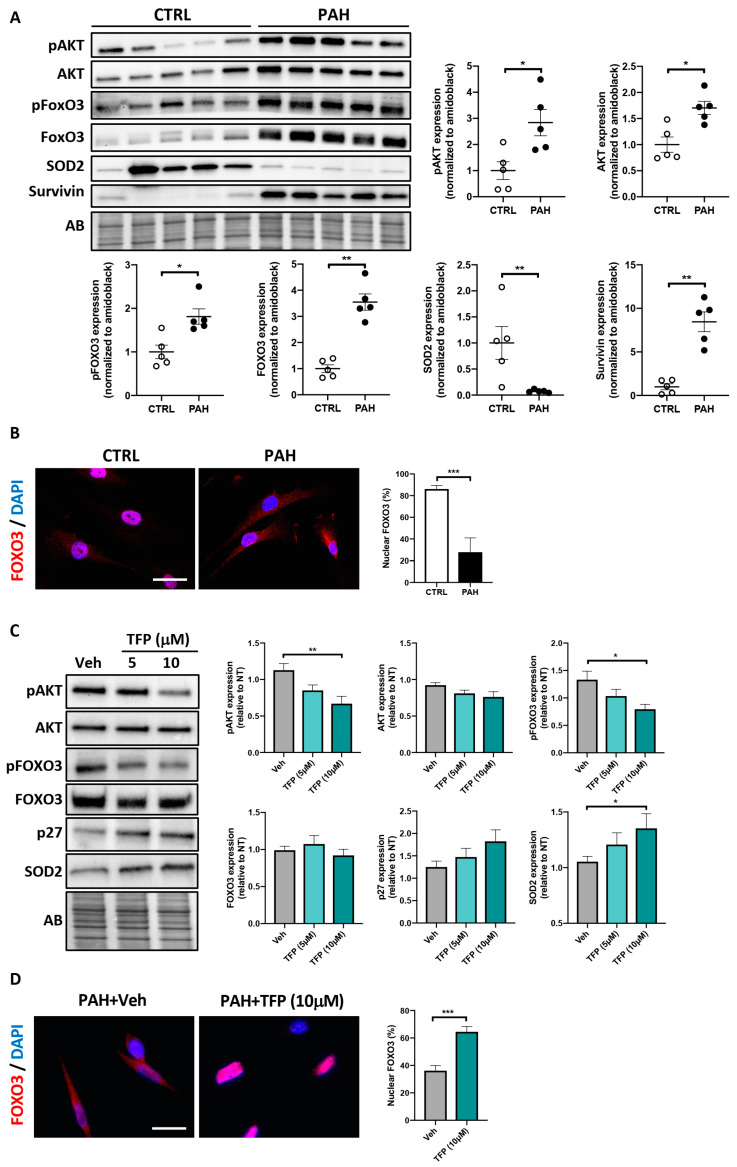
Trifluoperazine (TFP) inhibits AKT activity, leading to nuclear translocation of forkhead box protein O3 (FOXO3). (**A**) Representative Western blots and corresponding densitometric analyses of p(Ser473)-AKT, AKT, p(Ser253)-FOXO3, FOXO3, superoxide dismutase 2 (SOD2) and Survivin in PASMCs isolated from control (*n* = 5) and PAH (*n* = 5) patients. (**B**) Representative immunofluorescence images for subcellular localization of FOXO3 in cultured control and PAH-PASMCs. Quantification of the percentage of cells exhibiting nuclear expression of FOXO3 is shown. (**C**) Representative Western blots and corresponding densitometric analyses of p(Ser473)-AKT, AKT, p(Ser253)-FOXO3, FOXO3, p27 and SOD2 in PAH-PASMCs (*n* = 9) exposed or not to TFP for 24 h. Data are expressed as fold change relative to untreated cells. (**D**) Representative immunofluorescence images for subcellular localization of FOXO3 in PAH-PASMCs (*n* = 9) exposed to TFP (10 μM) or its vehicle for 24 h. Quantification of the percentage of cells exhibiting nuclear expression of FOXO3 is shown. Protein expression was normalized to Amido black (AB). * *p* < 0.05; ** *p* < 0.01 and *** *p* < 0.001. Scale bars: 20 μm.

Before investigating whether, as observed in cancer cells [12], TFP impacts AKT/FOXO3 signaling, we first determined whether increased AKT/FOXO3 signaling is a feature of the hyperproliferative and apoptosis-resistant PAH-PASMCs. To this end, we first measured expression levels of phosphorylated (activated) AKT (Ser473), AKT-dependent phosphorylation of FOXO3 (Ser253) and their respective total forms in the total lysates obtained from control and PAH-PASMCs. In agreement with the published data [20,21], the PAH-PASMCs displayed a distinct expression signature with an upregulation of Survivin and a marked diminution of the mitochondrial antioxidant enzyme superoxide dismutase 2 (SOD2) (Figure 2A). Furthermore, significantly higher levels of AKT and FOXO3 as well as their phosphorylated forms were detected in PAH-PASMCs compared to their normal counterparts (Figure 2A). To complement our findings, the subcellular localization of endogenous FOXO3 was next examined by immunofluorescence, using DAPI staining to define the nuclear area. As expected, PAH-PASMCs exhibited a prominent cytosolic localization of FOXO3, whereas FOXO3 was mainly distributed in the nucleus in control cells (Figure 2B). Exposure of PAH-PASMCs to TFP significantly decreased the levels of phosphorylated AKT and FOXO3, which was accompanied by a higher proportion of cells exhibiting nuclear localization of FOXO3 (Figure 2C,D). In agreement with these findings, TFP significantly impacted well-recognized FOXO3 downstream targets, with upregulation of the cell cycle inhibitor p27Kip1 and the antioxidant enzyme SOD2 (Figure 2C) [22,23].

### 2.3. Forced Nuclear Localization of FOXO3 Reduces PAH-PASMC Proliferation

**Figure 3:**
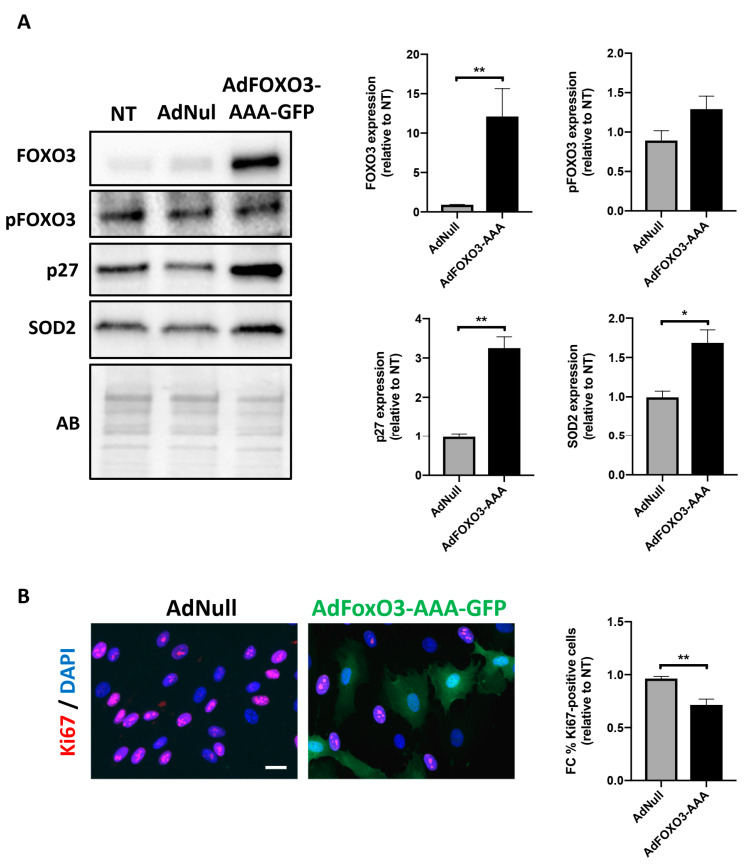
Forced expression of an adenovirus expressing a constitutively active form of FOXO3 reduces PAH-PASMC proliferation. (**A**) Representative Western blots and corresponding densitometric analyses of FOXO3, p(Ser253)-FOXO3, p27 and SOD2 in PAH-PASMCs (*n* = 5) infected or not with an adenovirus coding for a non-phosphorylable and constitutively active form of FOXO3 (AdFOXO3-AAA) or an empty adenovirus (AdNull) for 24 h. (**B**) Representative immunofluorescence images of PAH-PASMCs (*n* = 5) labeled with Ki67 after infection with AdFOXO3-AAA or AdNull for 24 h. Quantification of the percentage of PAH-PASMCs exhibiting nuclear expression of Ki67 is shown. Data are expressed as fold change (FC) relative to untreated cells and presented as mean ± SEM. Protein expression was normalized to Amido black (AB). * *p* < 0.05 and ** *p* < 0.01. Scale bars: 20 μm.

To investigate the relationship between FOXO3 expression/phosphorylation status and cellular effects, PAH-PASMCs were infected with an adenovirus expressing a constitutively active form of FOXO3 coupled to GFP (AdFOXO3-AAA, construct lacking the three AKT phosphorylation sites for nuclear export). After 48 h of adenoviral infection, FOXO3 overexpression was monitored by Western blot. A robust increase in FOXO3 expression without any impact on its phosphorylation level was detected, indicative of the effectiveness of infection (Figure 3A). Cell proliferation was next assessed by Ki67 labeling. After infection with constitutively active FOXO3a for 24 h, the number of Ki67-positive PAH-PASMCs was significantly diminished (Figure 3B). Consistently, an upregulation of p27 and SOD2 was observed in PAH-PASMCs overexpressing the FOXO3-AAA construct (Figure 3A).

### 2.4. Trifluoperazine Induces Autophagy

**Figure 4:**
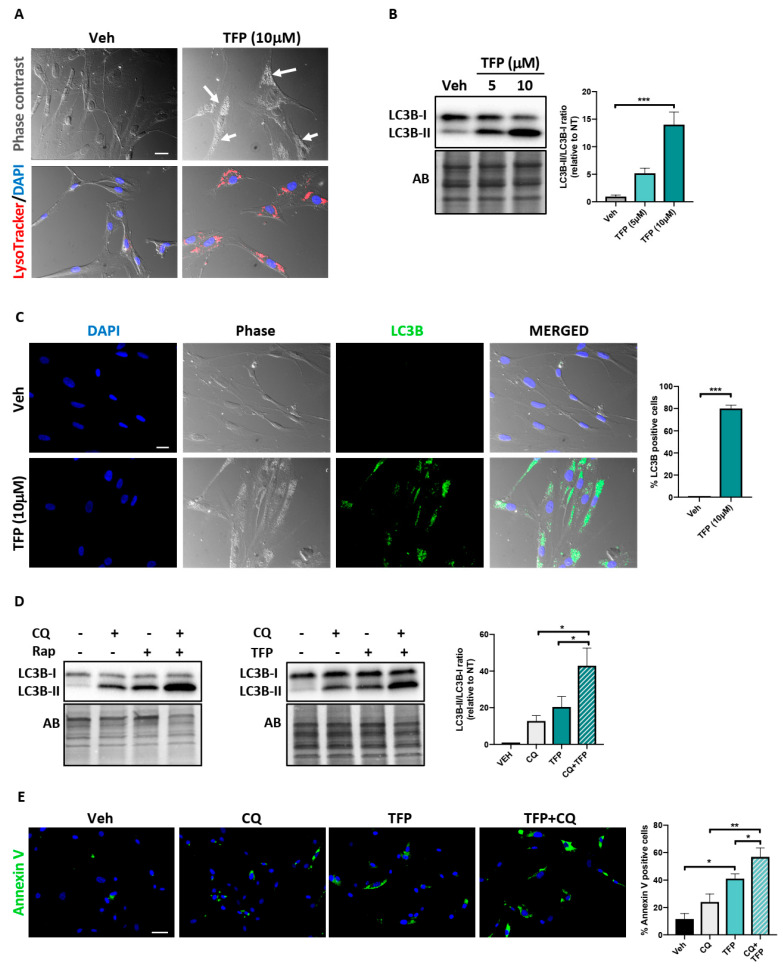
Induction of autophagy by trifluoperazine (TFP). (**A**) Bright-field images of PAH-PASMCs stained or not with LysoTracker Red after treatment or not with TFP (10 μM) for 48 h. Nuclei were counterstained with DAPI (blue). Arrowheads indicate cytoplasmic vacuolization. (**B**) Representative Western blot and corresponding densitometric analyses of light chain 3 (LC3)B in PAH-PASMCs exposed or not to TFP for 48 h. (**C**) Representative immunofluorescence images and corresponding quantification of PAH-PASMCs labeled with LC3B after treatment or not with TFP (10 μM). (**D**) Representative Western blot and corresponding densitometric analyses of LC3B in PAH-PASMCs exposed or not to rapamycin (Rap, 200 nM) or TFP (10 μM) for 48 h in presence or not to chloroquine (CQ, 50 μM) during the last 3 h. (**E**) Representative immunofluorescence images and corresponding quantification of apoptosis (as determined by Annexin V labeling) in PAH-PASMCs (*n* = 3) pretreated or not with CQ (10 μM) for 3 h before exposure or not to TFP (5 μM) for 24 h. Experiments were performed on three different PAH-PASMC cell lines. Data are presented as mean ± SEM; * *p* < 0.05; ** *p* < 0.01 and *** *p* < 0.001. Scale bars: 20 μm in A and C; 50 μm in E.

Exposure to TFP or its derivatives was previously reported to result from either induction of autophagy [11,24] or interruption of autophagy flux [19,25], leading to opposite cellular outcomes. To determine whether TFP influences autophagy, PAH-PASMCs were first observed under a bright-field phase-contrast microscope. TFP-treated PAH-PASMCs exhibited an obvious accumulation of small and large vesicle-like structures within the cytoplasm, a feature typically observed in cells undergoing autophagy (Figure 4A). These vesicles were positively stained with Lysotracker, suggesting them to be autolysosomes (Figure 4A). During autophagy, conversion of soluble microtubule-associated protein 1 light chain 3 (LC3-I) to lipid-bound LC3-II is associated with the formation of autophagosomes, which then fuse with lysosomes to form autolysosomes. As a result, the cytoplasmic material engulfed in autophagosomes is degraded by lysosomal hydrolytic enzymes [26,27]. We thus examined changes in the level of LC3 by Western blot. As shown in Figure 4B, treatment with TFP led to a dose-dependent increase in LC3-II level, indicating an accumulation of autophagosomes. This was confirmed by immunofluorescence, with a massive accumulation of cytoplasmic LC3-positive dots upon TFP exposure (Figure 4C).

The accumulation of autophagosomes in response to TFP treatment could result from autophagy induction or its blockade [26]. To further evaluate the effects of TFP on autophagic flux, we treated PAH-PASMCs with TPF in the presence of chloroquine (CQ, a well-established end-stage autophagic flux inhibitor blocking the fusion of autophagic vesicles and lysosomes) and examined the effect on LC3 expression. If TFP is an inducer of autophagic flux, combined treatment with a saturated inhibitor such as chloroquine should further increase the LC3-II/LC3-I ratio. Otherwise, combined treatment of TFP and CQ should not exert an additive effect. As observed after co-treatment with the autophagy inducer rapamycin and CQ, co-exposure of TPF and CQ led to a significantly higher level of LC3-II as compared with TFP or CQ alone (Figure 4D), indicating that TFP promotes autophagic flux. To investigate whether this autophagy induction played a protective or cytotoxic role, PAH-PASMCs were pre-treated, or not, with CQ (10 μM) for 3 h before being exposed to a low dose of TFP (5 μM) for 24 h. As shown in Figure 4E, TFP treatment induced an increase in apoptosis rate, and pre-treatment with CQ further increased the apoptosis rate compared to TFP alone, suggesting that induction of autophagy represents a failed attempt to cope with TFP-induced stress.

### 2.5. Trifluoperazine Significantly Improves Established PAH in the Sugen/Hypoxia (Su/Hx) Rat Model

**Figure 5:**
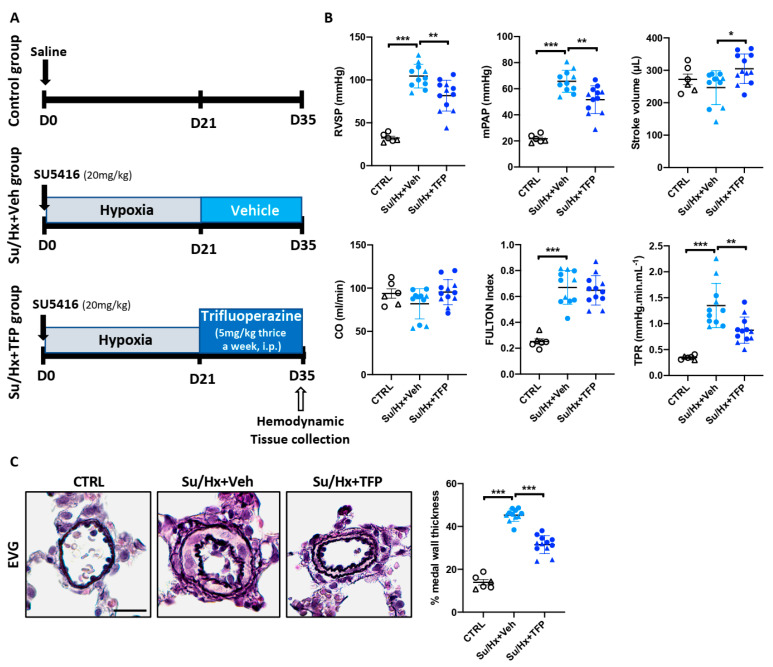
Effects of trifluoperazine (TFP) treatment on sugen/hypoxia (Su/Hx)-induced PAH in rats. (**A**) Schematic representation of the experimental design and schedule of the protocol. (**B**) Right ventricular systolic pressure (RVSP), mean pulmonary artery pressure (mPAP), stroke volume (SV), cardiac output (CO), Fulton index and total pulmonary resistance (TPR) were measured in control (CTRL), Su/Hx + vehicle (Veh) and Su/Hx + TFP rats; *n*= 6 to 12 rats/group. (**C**) Representative images of distal pulmonary arteries (PAs) stained with Elastica van Gieson (EVG). The graph on the right represents the degree of medial wall thickness. Data are presented as mean ± SEM and triangles represent females. * *p* < 0.05; ** *p* < 0.01; *** *p* < 0.001. Scale bars: 20 μm.

The anti-proliferative and anti-survival effects of TFP on cultured PAH-PASMCs prompted us to examine whether TFP can reverse established PAH in vivo. To this end, we first used the sugen/hypoxia (Su/Hx) rat model. For this purpose, male and female rats received a single injection of sugen (a vascular endothelial growth factor (VEGF) receptor inhibitor) and were then placed in hypoxia for 3 weeks before being randomly assigned to treatment with vehicle or TFP, thrice a week, for the subsequent 14 days (Figure 5A). As expected, injection of sugen followed by 3 weeks of hypoxia caused a substantial elevation of the right ventricular (RV) systolic pressure (RVSP) and mean PA pressure (mPAP), as assessed by right heart catheterization in closed-chest animals. Administration of TPF significantly reduced RVSP and mPAP compared to vehicle-treated Su/Hx rats (Figure 5B). Treatment with TFP also resulted in an increase in stroke volume, whereas only a non-significant tendency was noted for cardiac output (Figure 5B). As assessed by the Fulton index, there was no difference in RV hypertrophy among TFP- and vehicle-treated groups. Total pulmonary resistance (TPR, calculated as the ratio of RVSP to CO) was significantly reduced in Su/Hx rats receiving TFP (Figure 5B). Accordingly, we found that the medial wall thickness of distal PAs (<75 μm in diameter) was markedly attenuated upon treatment with TFP (Figure 5C). Improvement of pulmonary vascular remodeling was linked with diminished PASMC proliferation and augmented apoptosis in distal PAs along with more frequent nuclear FOXO3 staining (Supplementary Figures S1 and S2).

### 2.6. Beneficial Effects of Trifluoperazine in the Monocrotaline (MCT) PAH Rat Model

**Figure 6:**
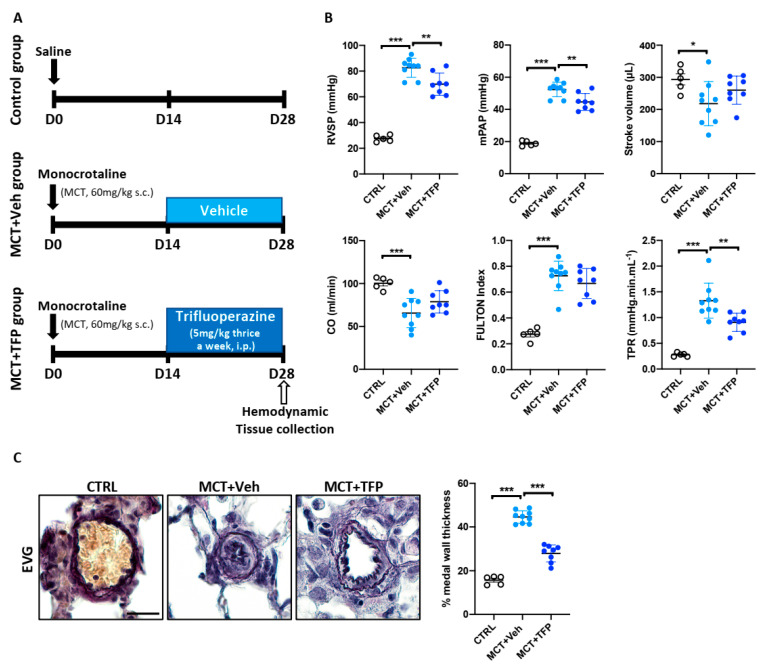
Effects of trifluoperazine (TFP) treatment on monocrotaline (MCT)-induced PAH in rats. (**A**) Schematic representation of the experimental protocol for induction and therapeutic intervention in the MCT-induced PAH rat model. (**B**) Right ventricular systolic pressure (RVSP), mean pulmonary artery pressure (mPAP), stroke volume (SV), cardiac output (CO), Fulton index and total pulmonary resistance (TPR) were measured in control (CTRL), MCT + vehicle (Veh) and MCT + TFP rats; *n*= 5 to 9 rats/group. (**C**) Representative images of distal pulmonary arteries (PAs) stained with Elastica van Gieson (EVG). The graph on the right represents the degree of medial wall thickness. Data are presented as mean ± SEM; * *p* < 0.05; ** *p* < 0.01; *** *p* < 0.001. Scale bars: 20 μm.

In order to strengthen our results and keeping in mind that PAH animal models do not recapitulate all aspects of human disease, we investigated the therapeutic potential of TFP in a second PAH animal model. For this purpose, male rats received a single injection of monocrotaline (MCT). Two weeks after MCT injection, rats were randomly divided into two groups that were treated with either TFP or vehicle (Figure 6A). Consistent with the results obtained in the Su/Hx-induced PAH model, administration of TFP significantly lowered RVSP and mPAP (Figure 6B). Although TFP did not attenuate RV hypertrophy (Figure 6B), stroke volume (SV) and cardiac output (CO), which decreased in vehicle-treated Su/Hx animals compared to controls, exhibit a tendency to increase with TFP treatment (Figure 6B). Furthermore, the TPR and medial wall thickness of distal PAs were significantly diminished in MCT rats treated with the compound (Figure 6C). As expected, proliferation and apoptosis of distal PASMCs were reduced and increased, respectively, in TFP-treated rats (Supplementary Figure S1). These effects were accompanied by an enhanced FOXO3 nuclear localization (Supplementary Figure S2). Altogether, these results indicate that TFP provides therapeutic benefits in two preclinical PAH animal models without causing detectable side effects.

## 3. Discussion

Despite research advances and the availability of approved drugs, PAH remains an incurable disease with high morbidity and early mortality. Drug repositioning/repurposing has gained increased attention as an alluring (i.e., cost-effective, accelerated and effective) strategy for finding new therapeutic options [9]. Based on the literature signals showing that TFP, an approved antipsychotic drug, elicits anti-tumoral effects in experimental models by targeting various pathways already known to promote pulmonary vascular remodeling in PAH, we investigated its therapeutic efficacy both in vitro and in vivo. In the present work, we showed that TFP diminishes the pro-survival and hyper-proliferative capacity of cultured PAH-PASMCs. At the mechanistic level, TFP was found to reduce activation of the multitasking kinase AKT, leading to nuclear translocation of FOXO3 and proliferation slow-down. Furthermore, we provide evidence that TFP induces autophagy as an unsuccessful attempt to promote cell survival. More importantly, we demonstrate that administration of TFP significantly reduces PA remodeling and improves hemodynamic parameters in two preclinical animal models of PAH.

In agreement with published data derived from the cancer field, several studies have highlighted the serine/threonine kinase AKT as a pivotal point of converging signaling pathways involved in pulmonary vascular remodeling. Indeed, overactivated in response to growth factors released by stressed PA resident cells, AKT was reported to regulate the activity of multiple downstream targets, which, in turn, act synergistically to enhance PASMC proliferation and survival [8,28,29]. Among its well-documented effects in PAH cells, AKT was reported to promote phosphorylation and nuclear exclusion of FOXO1, thereby alleviating its transcriptional repressive effects on mitogenic and anti-apoptotic genes [30]. Similarly to that observed in breast cancer cells [11,12], treatment of PAH-PASMCs with TFP led to a significant downregulation of AKT activity. Moreover, this effect was associated with nuclear shuttling of FOXO3 and subsequent induction of anti-proliferative genes. Contrary to its paralog FOXO1 [30], the implication of FOXO3, a well-established tumor suppressor with regular overlap and functional redundancy [31], has not received particular attention in PAH. We found that PAH-PASMCs exhibit elevated levels of phosphorylated FOXO3 and accordingly prominent nuclear exclusion of FOXO3. The importance of AKT-dependent cytosolic localization of FOXO3 in the cancer-like phenotype of PAH-PASMCs was further highlighted by forced expression of a FOXO3 mutant lacking the AKT phosphorylation sites. Indeed, reduced PAH-PASMC proliferation was observed upon transfection, supporting the notion that, in addition to FOXO1, FOXO3 inactivation/nuclear exclusion contributes to the hyperproliferative state of PAH-PASMCs.

Autophagy is a stress-responsive, dynamic and cellular self-catabolic process in which intracellular proteins and organelles are degraded and recycled to supply fundamental building blocks to maintain energy homeostasis [27]. By doing so, autophagy is considered to confer stress tolerance/resistance, limit damage and, thus, sustain cell survival under adverse conditions. Accordingly, blocking autophagic response has been shown to induce or potentiate cell death induced by various anti-tumor agents. However, in numerous circumstances, autophagy functions as a double-edged sword as both excessive or insufficient levels of autophagic flux can affect cell viability and precipitate cell death [32]. Although TFP was reported to exert anti-proliferative and anti-survival effects in multiple cancer models [12,18,19,25,33], conflicting data exist regarding the impact of TFP on autophagy and its consequence on cell viability [19,24,34,35]. Herein, we showed that anti-proliferative and anti-survival effects of TFP were accompanied by induction of autophagy. Our further experiments indicate that treatment of PAH-PASMCs with CQ or TFP alone induces PAH-PASMC cell death and that the combination of both amplifies this effect. These results strengthen published data showing that CQ inhibits proliferation and stimulates apoptosis of PASMCs [36] and suggest that enhanced autophagy secondary to TFP exposure represents an unsuccessful protective mechanism.

Interestingly, various mechanisms seem to account for the anti-tumor effects of TFP [11,12,13,14,15,18]. In addition to inhibiting calmodulin and downregulating AKT activity, TFP was reported to inhibit DNA repair efficiency in cancer cells by decreasing expression of several DNA repair proteins including RAD51 [19,37,38], found to be upregulated in PAH cells [39]. Since PAH-PASMCs rely on an efficient DNA damage response to sustain their survival and proliferation under persistent stress conditions [39,40], it is tempting to speculate that the death of PAH-PASMCs induced by TFP also results from interference with the DNA repair machinery. Similarly, we cannot exclude the fact that the anti-adrenergic and anti-dopaminergic actions of TFP account for its beneficial effects on the pulmonary vasculature. Finally, it must be emphasized that therapeutic interventions aiming at reversing established pulmonary vascular remodeling are considered potentially cardiotoxic. Although RV hypertrophy was unchanged after TFP treatment in animal models (possibly due to insufficient treatment duration), examination of RV function by right heart catheterization revealed no deleterious or even salutary effects. Consistently, TFP was shown to improve regional myocardial function after acute coronary artery occlusion [41].

Regardless of the exact mechanisms by which TFP exerts its anti-survival and anti-proliferative effects on PAH-PASMCs, the present study provides evidence that its administration improved established PAH in two preclinical models, which supports the view that seeking new uses for old drugs may represent a fruitful approach.

## 4. Materials and Methods

### 4.1. Isolation and Culture of Human Pulmonary Arterial Smooth Muscle Cells

PAH-PASMCs were isolated from small pulmonary arteries (<1000 μm diameter) from 11 PAH patients, all diagnosed and managed according to recent guidelines. Control PASMCs (*n* = 7 cell lines) were either purchased from Cell Application or isolated from patients without PAH at autopsy. Clinical and hemodynamic characteristics of patients are shown in Supplemental Table S1. PASMCs were grown in high-glucose Dulbecco’s Modified Eagle’s medium (DMEM) supplemented with 10% fetal bovine serum (FBS). The purity of the PASMCs in the primary cultures was confirmed by staining for alpha smooth muscle actin (αSMA) using the immunofluorescence technique. Only cells between passages 4 and 8 were used for experiments.

### 4.2. Cell Culture and Treatments

Trifluoperazine (TFP), chloroquine (CQ) and rapamycin (Rap) were purchased from MilliporeSigma (Oakville, ON, Canada), dissolved in dimethyl sulfoxide (DMSO) and then added to the culture medium at the indicated concentrations immediately before use. Replication-defective adenoviral vectors encoding a constitutively active mutant form of FOXO3a (Ad-AAA-O3a) and co-expressing green fluorescent protein (GFP) as well as empty adenoviral vectors (AdNull) were purchased from Vector Biolabs. Cells were infected at 1 × 10^7^ plaque-forming units (pfu)/mL for 24 h before harvesting and analysis.

### 4.3. Proliferation and Apoptosis Assay

To assess cell proliferation and apoptosis, PAH-PASMCs were cultured for 24 h in 10% fetal bovine serum. Cell proliferation was determined with either Ki67 labeling or 5-Ethynyl-2′-deoxyuridine (EdU) incorporation assay (Click-iT EdU assay kit, Thermo Fischer Scientific, Ottawa, ON, Canada) according to the manufacturer’s instructions. Briefly, EdU was added during the last 2 h. After incubation, EdU-positive DNA-duplicating cells were fixed with 3.7% formaldehyde diluted in phosphate-buffered saline (PBS) 1X for 15 min at room temperature, washed with PBS 1X and then permeabilized for 20 min in 0.5% Triton X-100 in PBS. After washing in 3% BSA in PBS 1X, cells were stained with the Click-iT reaction mix for 30 min and counterstained with DAPI. Apoptosis was evaluated by Annexin V assay, as previously described [20,42]. The Ki67/EdU proliferative and Annexin V apoptotic index were calculated by counting the number of positive-staining cells divided by the total number of DAPI-positive cells multiplied by 100. For each cell line, experiments were performed in triplicate and at least 300 cells per condition were counted.

### 4.4. Western Blotting

Proteins from cultured PAH-PASMCs were extracted using a 2% Chaps protein extraction buffer supplemented with a protease-inhibitor cocktail (Roche, Laval, QC, Canada). Lysate total protein concentration was determined using the Bradford method. Equal amounts of protein (10–20 µg) were resolved on SDS-polyacrylamide gels and transferred to polyvinylidene fluoride or nitrocellulose membranes using a semi-dry transfer system (Bio-Rad Laboratories, Mississauga, ON, Canada). Membranes were subsequently blocked with either 5% goat serum, 5% non-fat dry milk or 5% bovine serum albumin for 1 h before being incubated overnight at 4 °C with indicated primary antibodies (Supplemental Table S2). After being rinsed 3 times with TBS-Tween 0.1%, membranes were incubated with appropriate horseradish peroxidase (HRP)-conjugated secondary antibody for 1 h at room temperature. Antibodies were revealed using enhanced chemiluminescence (ECL) reagents (Perkin–Elmer, Woodbridge, ON, Canada) and labeled proteins were detected with the imaging Chemidoc MP system (Bio-Rad Laboratories, Mississauga, ON, Canada). Protein expression was quantified using the Image lab software (Bio-Rad Laboratories) and normalized to Amido black (AB) as previously described [42].

### 4.5. Animal Models

All animal protocols were approved by the Laval University and the Institut Universitaire de Cardiologie et de Pneumologie de Québec (IUCPQ) Biosafety and Ethics Committees (#2019-311). All experiments were in accordance with recent recommendations on optimal preclinical studies in PAH, including animal randomization, sequence allocation concealment and blinded assessments [43,44]. Sugen-hypoxia (Su/Hx) and monocrotaline (MCT) PAH rat models were used in the present study. For the Su/Hx model, both adult male and female Sprague Dawley rats (Charles River Laboratories, Montreal, QC, Canada) were injected with 20 mg/kg of SU5416 (Sugen, MilliporeSigma, Oakville, ON, Canada) and put in hypoxia (10% O_2_) for 3 weeks. For the MCT model, rats were injected subcutaneously with 60 mg/kg of monocrotaline (MilliporeSigma, Oakville, ON, Canada). As females are traditionally considered as more resistant than males to developing MCT-induced pulmonary hypertension, only males were used. Once PAH was established (at days 14 and 21 post-MCT or -SU5416 injection, respectively), rats were randomly divided into two groups and received trifluoperazine (5 mg/kg delivered intraperitoneally, three times a week) or its vehicle for 2 additional weeks.

### 4.6. Hemodynamic Measures of RV Function and Assessment of RV Hypertrophy and PA Wall Thickness

Before sacrifice, rats were initially anesthetized with 3–4% isoflurane and maintained with 2% during procedures. Hemodynamic parameters including RV systolic pressure (RVSP), stroke volume, cardiac output (CO) and total pulmonary resistance (TPR) were measured blindly by right heart catheterization (SciSence catheters) in closed-chest animals, as previously described [20,45]. The mean PA pressure was estimated using the following formula: mPAP = 0.61*RVSP + *2* mmHg. After sacrifice, the heart was excised and the RV free wall was separated from the left ventricle and interventricular septum (LV+IVS). As a surrogate of RV hypertrophy, the ratio of RV weight to LV+IVS weight (Fulton’s index) was calculated. Morphometric analysis of PA medial wall thickness was performed as previously described [20,39]. Briefly, paraffin-embedded 5-µm-thick lung sections were stained with Elastica van Gieson (EVG). The medial wall thickness was calculated in at least 15 randomly selected distal PAs (<75 µm in diameter) per animal with the following formula: 100 × (external diameter − internal diameter)/external diameter.

### 4.7. Immunohistochemistry

Paraffin-embedded lungs were serially sectioned at 5 µm. Lung sections were dewaxed and rehydrated in graded ethanol solutions. Once rehydrated, slides were subjected to antigen retrieval in citrate buffer (0.01 M, pH 6.0) in a microwaveable pressure cooker for 20 min. Sections were blocked with 5% goat serum for 3 h and then incubated with indicated primary antibodies in a humidified chamber overnight at 4 °C. After washes, sections were further incubated for 1 h at room temperature with appropriate fluorescent dye-conjugated secondary antibodies (Table S2). Sections were mounted onto coverslips using DAPI (4′,6-diamidino-2-phenylindol) Fluoromount G mounting medium. Sections were examined by microscopy using an Axio Observer microscope (Carl Zeiss, North York, ON, Canada), and images were acquired using Zen system (Carl Zeiss, North York, ON, Canada).

### 4.8. Statistical Analyses

All analyses were performed using GraphPad Prism 6.0 (GraphPad, San Diego, CA, USA). The unpaired Student *t*-test and one-way analysis of variance (ANOVA) test were used for comparisons between 2 and 2 or more normally distributed groups, respectively. The Mann–Whitney and Kruskal–Wallis non-parametric tests were used to compare 2 or more non-normally distributed groups. A significance level inferior to 5% (*p* < 0.05) was considered statistically significant.

## Data Availability

The data that support the findings of this study are available from the corresponding author upon reasonable request.

## Supplementary Materials

The following are available online at https://www.mdpi.com/1422-0067/22/6/2919/s1.
