# Supplementary material for: Preclinical Investigation of Trifluoperazine as a Novel Therapeutic Agent for the Treatment of Pulmonary Arterial Hypertension
Source: Int J Mol Sci. 2021 Mar 13;22(6):2919. doi: 10.3390/ijms22062919 (PMC7998101; doi:10.3390/ijms22062919)
Supplement: Supplementary file 1 [file ijms-22-02919-s001.pdf]

Supplemental Data

Title: Preclinical Investigation of Trifluoperazine as a Novel Therapeutic Agent for The Treatment of Pulmonary Arterial Hypertension.

Authors: Yann Grobs[1], Charifa Awada[1], Sarah-Eve Lemay[1], Charlotte Romanet[1], Alice Bourgeois[1], Victoria Toro[1], Valérie Nadeau[1], Kana Shimauchi[1], Mark Orcholski[1], Sandra Breuils-Bonnet[1], Eve Tremblay[1], Steeve Provencher[1,2], Roxane Paulin[1,2], Olivier Boucherat[1,2] and Sébastien Bonnet[1,2,#].

[1] Pulmonary Hypertension Research Group, Québec Heart and Lung Institute Research Centre, G1V4G5 Québec City, QC, Canada.
[2] Department of Medicine, Laval University, G1V4G5 Québec City, QC, Canada.

	CTRL (n=7)	PAH (n=11)
Age (years)	44.5±4.8	46.1±9.2
Gender (female (%))	1 (17%)	6 (54.5%)
PAH group		
- HPAH		2 (18.2%)
- IPAH		8 (72.7%)
-SSc-PAH		1 (9.1%)
Autopsy (n (%))	-	9 (81.8%)
Transplantation (n (%))	-	2 (18.2%)
Pulmonary hemodynamics		
mPAP (mmHg)	-	57.9±10
CO ($L.min^{-1}$)	-	5.3±1.5
PVR ($dyne.sec^{-1}.cm^{-5}$)	-	676±224
Medication		
Endothelin receptor antagonist	-	3 (27.3%)
PDE5 inhibitor	-	7 (63.6%)
Prostacyclin analog	-	3 (27.3%)
Unknown	-	4 (36.4%)

Table S1. Clinical characteristics of PAH patients and controls. Values are means ± SD. HPAH: heritable PAH; IPAH: idiopathic PAH; SSc-PAH: Systemic sclerosis-associated PAH; mPAP: mean pulmonary arterial pressure; CO: cardiac output; PVR: pulmonary vascular resistance; PDE5: phosphodiesterase-5. Note than some patients take more than one type of medication.

Target Antigen	Vendor	Catalog #	Concentration	Dilution
Western blot				
MCM2	Bethyl Laboratories	A300-191A (Rabbit polyclonal)	Not specified	1:1000
PLK1	Abcam	ab17056 (Mouse monoclonal, 35-206)	Not specified	1:1000
Survivin	Cell Signaling	#2808 (Rabbit monoclonal, 71G4B7)	620μg/ml	1:1000
p(Ser473)-AKT	Cell Signaling	#9271 (Rabbit polyclonal)	10μg/ml	1:1000
AKT	Cell Signaling	#9272 (Rabbit polyclonal)	35μg/ml	1:1000
p(Ser253)-FOXO3	Cell Signaling	#9466 (Rabbit polyclonal)	900μg/ml	1:1000
FOXO3	Cell Signaling	#2497 (rabbit monoclonal, 75D8)	15μg/ml	1:1000
SOD2	Millipore Sigma	06-984 (Rabbit polyclonal)	1mg/ml	1:1000
P27	Cell Signaling	#3698 (Mouse monoclonal, SX53G8.5)	1mg/ml	1:1000
LC3B	Cell Signaling	#3858 (Mouse monoclonal, D11)	500μg/ml	1:1000
P62/SQSTM1	Cell Signaling	#5114 (Rabbit polyclonal)	20μg/ml	1:1000
Immunofluorescence				
Ki67 (ICF)	Millipore Sigma	AB9260 (rabbit polyclonal)	Not specified	1:300
FOXO3 (ICF % IHF)	Cell Signaling	#12829 (Rabbit monoclonal, D19A7)	130μg/ml	1:400
LC3B (ICF)	Cell Signaling	#3858 (Mouse monoclonal, D11)	500μg/ml	1:200
PCNA (IHF)	Agilent-Dako	M0879 (Mouse monoclonal, PC10)	330μg/ml	1:400
Cleaved Caspase-3 (IHF)	Cell Signaling	#9661 (Rabbit polyclonal)	52μg/ml	1:400
αSMA (IHF)	Sigma-Aldrich	#A2547 (Mouse monoclonal,1A4)	Not specified	1:200
αSMA (IHF)	Abcam	Ab5694 (Rabbit polyclonal)	200μg/ml	1:200

Table S2: Primary antibodies used for Western blotting and immunofluorescence analysis. ICF: immunocytofluorescence; IHF: immunohistofluorescence

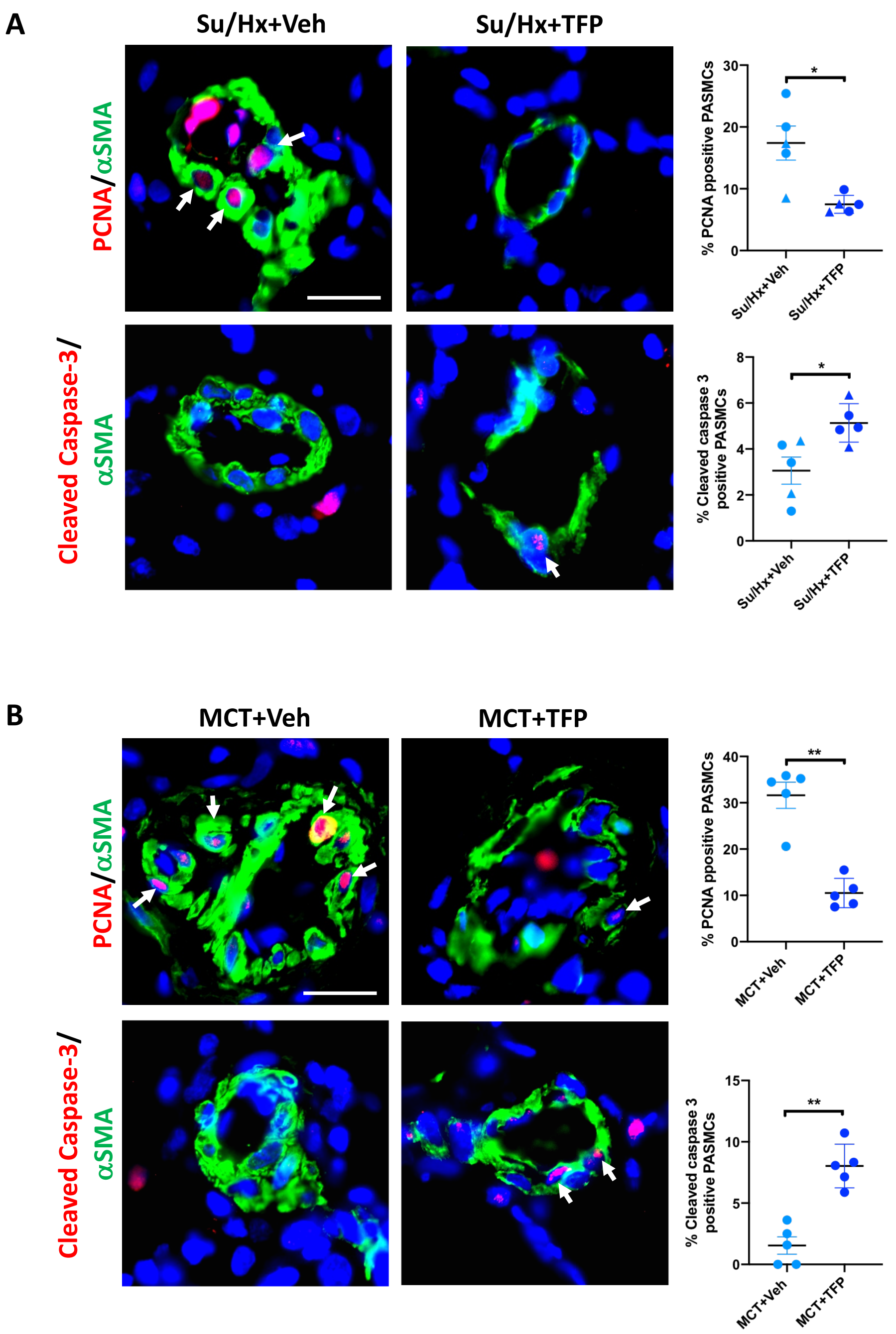

Figure S1: Impact of Trifluoperazine (TFP) on PASMC proliferation and apoptosis in PAH animal models. Proliferation and apoptosis were studied in lungs of Su/Hx+Vehicle, and Su/Hx+TFP (A) or MCT+Vehicle, and MCT+TFP (B) rats. Representative images of distal pulmonary vessels labeled with proliferating cell nuclear antigen (PCNA) or Cleaved Caspase-3 in red. Vascular smooth muscle cells were labeled using alpha smooth muscle actin (αSMA, green). Graphs on the right represent the percentage of PASMCs positive for PCNA- or Cleaved Caspase-3 in distal pulmonary vessels. Arrows mark positive cells. Data are presented as mean±SEM and triangles represent females. n = 5 rats/group (mean of 15 vessels/rat). *$P < 0.05$ and **$P < 0.01$. Scale bars = 20µm.

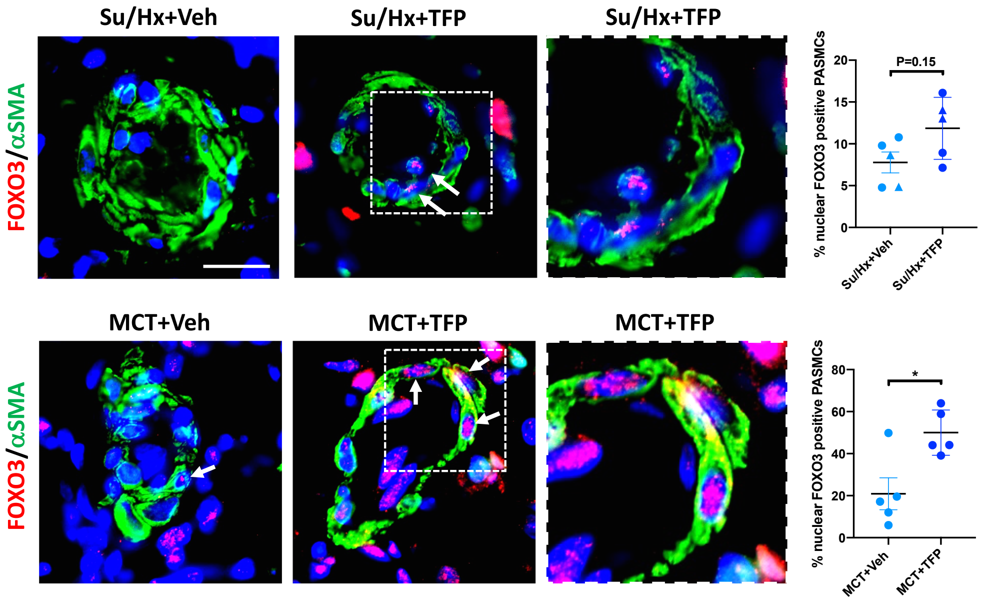

Figure S2: Impact of Trifluoperazine (TFP) on FOXO3 localization in vivo. Representative images of distal pulmonary vessels from Su/Hx and MCT rats exposed or not to TFP and labeled with FOXO3 (red). Vascular smooth muscle cells were labeled using alpha smooth muscle actin (αSMA, green). Graphs on the right represent the percentage of PASMCs presenting nuclear expression of FOXO3. Arrows mark PASMCs exhibiting nuclear expression of FOXO3. Data are presented as mean±SEM and triangles represent females. n = 5 rats/group (mean of 15 vessels/rat). *$P < 0.05$. Scale bars = 20µm.
